# Assessment of Suitable Habitats and Quality of *Siraitia grosvenorii* in China

**DOI:** 10.1002/ece3.72600

**Published:** 2025-11-29

**Authors:** Yang Yang, Mingli Hu, Leilei Yang, Jingyuan Wang, Tao Tian, Wenyang Qing, Shengmei Yang, Jun He, Qing Yang, Sisheng Zhang

**Affiliations:** ^1^ School of Pharmaceutical Sciences Hunan University of Medicine Huaihua China; ^2^ Hunan Provincial Key Laboratory for Synthetic Biology of Traditional Chinese Medicine Hunan University of Medicine Huaihua China; ^3^ School of Pharmacy, Xianning Medical College Hubei University of Science and Technology Xianning China; ^4^ Laboratory of Southern Subtropical Plant Diversity Fairylake Botanical Garden, Shenzhen & Chinese Academy of Sciences Shenzhen China; ^5^ School of Animal and Veterinary Sciences Southwest Minzu University Chengdu China; ^6^ Wenshan Academy of Agricultural Sciences Wenshan China

**Keywords:** bioactive ingredients, environmental variable, habitat suitability, in vitro antioxidant capacity, MaxEnt model, species distribution

## Abstract

Climate change impacts the habitats of medicinal plants and potentially affects the quality of herbal medicines. *Siraitia grosvenorii*, a crucial medicinal and edible traditional Chinese material endemic to China, requires more research on climate adaptation. This study employed Maxent and ArcGIS software to predict suitable habitats for *S. grosvenorii* across various periods in China. High‐Performance Liquid Chromatography and enzyme‐linked immunosorbent assay measured mogrosides V (MV) content, total flavonoid content (TFC), and total phenolic acid (TPA) in samples from different suitable habitats. Additionally, the in vitro antioxidant potency composite index (APCI) of various samples was compared. The results indicate that precipitation and temperature emerged as significant factors influencing the distribution of *S. grosvenorii*, with precipitation of warmest quarter (Bio_18), temperature seasonality (Bio_4), and precipitation of wettest quarter (Bio_16) identified as the key factors. Currently, suitable habitats for *S. grosvenorii* are primarily located south of the Yangtze River, especially in Guangxi and Guangdong Provinces. Future projections indicate a northward expansion of suitable habitats. MV content was significantly higher in samples from high‐ and medium‐suitability habitats compared to those from low‐suitability habitats. Conversely, TFC, TPA, and APCI values were higher in low‐suitability habitats. These findings offer valuable insights for identifying optimal cultivation areas and assessing the quality of *S. grosvenorii* resources in China.

## Graphical abstract

Climate change affects the habitats and quality of *Siraitia grosvenorii*, a key Chinese medicinal plant. This study used Maxent and ArcGIS to predict its suitable habitats and found that precipitation and temperature (especially Bio_18, Bio_4, and Bio_16) are key distribution factors, with current habitats mainly south of the Yangtze River and future expansion northward. Higher mogrosides V were found in high/medium‐suitability habitats, while total flavonoid, phenolic acid, and antioxidant capacity were higher in low‐suitability habitats, offering insights for cultivation and quality assessment.

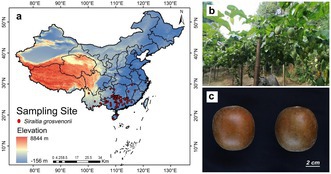

## 1 Introduction

*Siraitia grosvenorii* (Swingle) C. Jeffrey ex A. M. Lu & Zhi Y. Zhang, commonly known as Luo‐Han‐Guo or Monk fruit, is an endemic perennial herbaceous vine in the Cucurbitaceae family native to China. Its dried fruits have been used as a traditional “Homologue of food and medicine” herb, often employed to treat cough, asthma, sore throat, constipation, and other ailments (China Pharmacopoeia Commission 2025; Guo et al. 2024). Modern medicine has demonstrated *S. grosvenorii*'s effectiveness in boosting immunity, protecting the liver, and exhibiting antidiabetic, antitumor, antioxidant, and anti‐aging properties (Nie et al. 2019; Gong et al. 2022; Guo et al. 2024; Huang et al. 2024; Wu et al. 2024). *S. grosvenorii* is a primary source material for over 20 traditional Chinese medicines (TCM) in China. Researchers have isolated 131 compounds from *S. grosvenorii* (Huang et al. 2024), with mogroside V (MV) being the major constituent. MV is also the active medicinal ingredient used for quality evaluation of herbal medicine as stipulated in the Pharmacopeia of the People's Republic of China (PPRC) (China Pharmacopoeia Commission 2025). MV possesses a remarkable sweetness, 425 times that of sucrose, while its caloric content is only 1/50th of sucrose (Li et al. 2014). It is non‐toxic, does not induce tooth decay, and is safe for consumption by diabetic individuals, making it a natural sweetener and a viable alternative to sucrose (Zhou et al. 2022). Consequently, *S. grosvenorii* has significant economic value as a medicinal and sweetener plant resource in China.

The earliest documentation of *S. grosvenorii* appears in the Chinese medical classic “Xiuren XianZhi” from the Qing Dynasty (Li, Fan, Zheng, et al. 2020). It is primarily distributed in the southern provinces of Guangxi and Guangdong in China, with Guangxi Province accounting for over 90% of the global cultivation area. Yongfu and Lingui counties, with a rich cultivation history of over 160 years, are recognized as the origin centers for *S. grosvenorii* cultivation (Wei et al. 2020). Driven by its economic potential, *S. grosvenorii* has been extensively introduced to other provinces in China, including Hunan, Guizhou, Jiangxi, Fujian, Yunnan, and Zhejiang. However, its cultivation requires stringent environmental conditions (Wei et al. 2020; Yang 2020). Irrational and unscientific introduction of Chinese medicinal plants to unsuitable regions can lead to drastic declines in yield and significant economic losses in these areas.

Global climate change significantly affects the migration and habitat range of medicinal plants, potentially threatening the sustainable use of these valuable resources. Utilizing climate data to construct species distribution models has become a common approach in research addressing climate change (Zhao et al. 2021; Zhan et al. 2022; Yang, He, et al. 2023; Li, Yang, Gu, et al. 2024; Li, Yang, Feng, et al. 2024; Li et al. 2025). The Maxent model (Columbia University, USA) is a quantitative analytical tool used to predict the potential geographical distribution and suitable habitats of species (Yang, He, et al. 2023). The Maxent model, known for its ease of operation and high accuracy, also performs excellently with small‐sample data; thus, it is currently one of the most effective models for predicting the probability distribution of target objects (Hernandez et al. 2006; Deb, Jamir, and Kikon 2017; Tafesse et al. 2023). This model assesses the relationship between species distribution and environmental variables such as precipitation, humidity, temperature, and soil characteristics (Li, Fan, and He 2020; Zhao et al. 2021; Zhan et al. 2022; Yang, He, et al. 2023). By estimating the probability distribution that maximizes entropy, the Maxent model predicts the probability distribution of the target organism, making it one of the most effective models for distribution prediction. With advancements in conservation biology, ecology, and related fields, the Maxent model's applications have expanded across various species, time periods, and research needs (Zhao et al. 2021; Zhan et al. 2022; Tafesse et al. 2023; Yang, He, et al. 2023). Environmental factors influence not only the distribution of plants but also the types and quantities of their secondary metabolites. Suitable habitats are both prerequisites and crucial determinants for the synthesis and accumulation of secondary metabolites in medicinal plants such as *Zanthoxylum nitidum*, *Swertia bimaculata*, and *Panax notoginseng* (Zhan et al. 2022; Yang, He, et al. 2023; Wang et al. 2025).

Recent research on *S. grosvenorii* has primarily concentrated on its chemical composition and pharmacological properties (Zhu et al. 2020; Gong et al. 2022), with other studies exploring its metabolomics and transcriptomics (Itkin et al. 2016; Tu et al. 2017). However, limited attention has been given to its suitable habitats, overall distribution, and quality evaluation. While Wei et al. (2020) conducted a preliminary global analysis of suitable habitats for *S. grosvenorii*, a detailed investigation of its suitable habitats under current and future climate scenarios in China remains absent. Moreover, literature evaluating the quality of *S. grosvenorii* grown in different suitable habitats is scarce.

High‐performance liquid chromatography (HPLC), a commonly used technique for quantifying chemical constituents in medicinal plants, offers high sensitivity and allows for rapid and efficient separation and detection of compounds present in a sample. This technique is instrumental in evaluating variations in secondary metabolites among medicinal plants cultivated in different regions (He et al. 2024; Yang, Li, et al. 2023). This study utilized the MaxEnt model and ArcGIS software to analyze suitable habitats for *S. grosvenorii* in China under different climate scenarios. HPLC and enzyme‐linked immunosorbent assay (ELISA) measured MV content, total flavonoid content (TFC), and total phenolic acid (TPA) in samples from different suitable habitats. Additionally, the in vitro antioxidant potency composite index (APCI) of various samples was compared. Through a comprehensive evaluation of climate conditions, bioactive ingredients, and pharmacological action, suitable cultivation areas for *S. grosvenorii* in mainland China were identified. These findings provide valuable insights for land resource management and the rational introduction of *S. grosvenorii*.

## 2 Materials and Methods

### 2.1 Species Occurrence Records and Sample Collection

**FIGURE 1:**
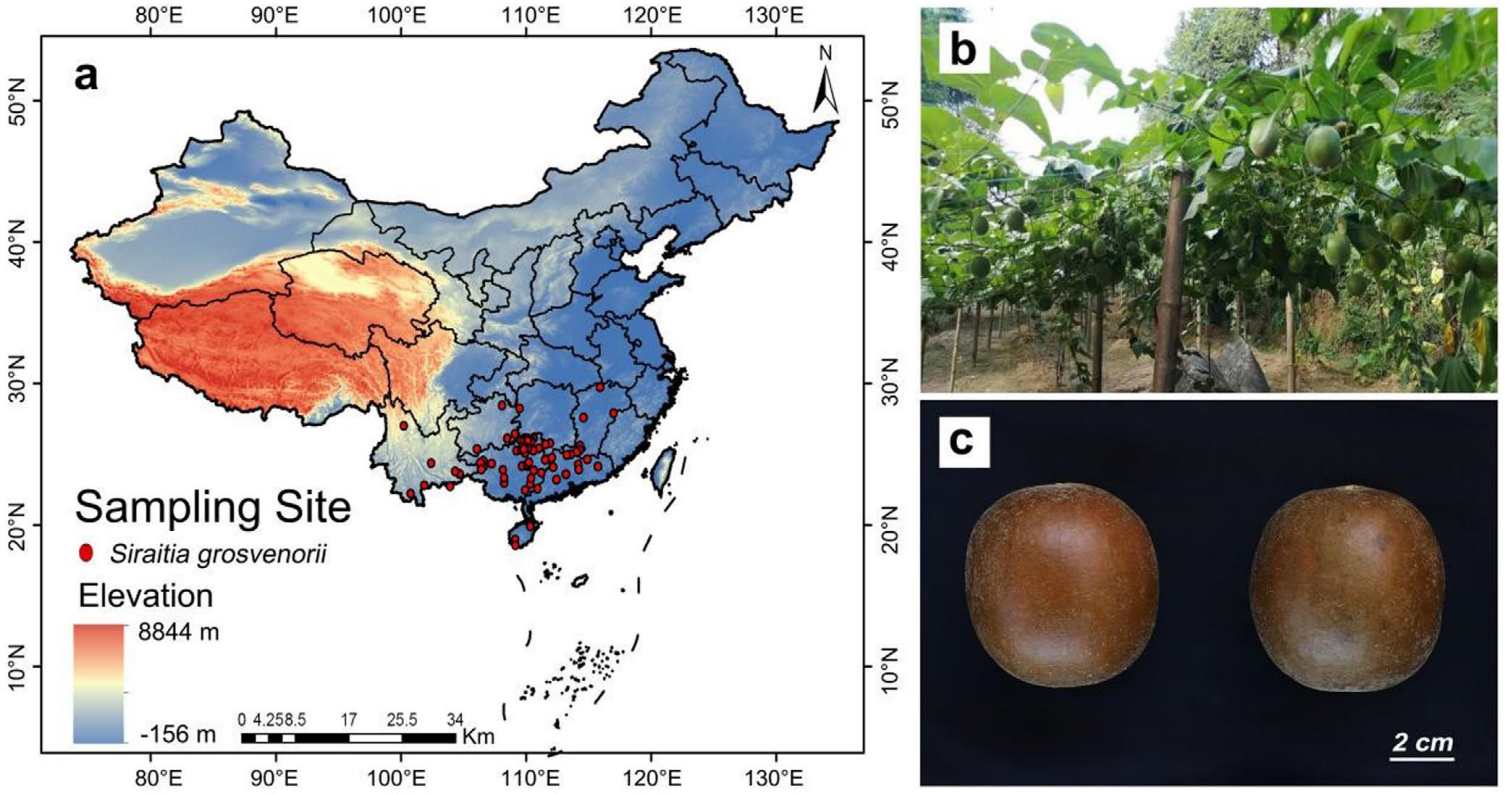
(a) Spatial distribution of *Siraitia grosvenorii* in China. (b) *S. grosvenorii* growing in a field. (c) Dried fruit of *S. grosvenorii* (scale bar = 2 cm).

Distribution data for *S. grosvenorii* was sourced from the National Specimen Information Infrastructure (NSII, http://www.nsii.org.cn/) and the Chinese Virtual Herbarium (CVH, http://www.cvh.ac.cn/). A total of 295 occurrence records from various provinces were gathered through online surveys: 127 in Guangxi, 43 in Jiangxi, 31 in Guangdong, 14 in Yunnan, 13 in Guizhou, 11 in Hainan, 7 in Fujian, and 4 in Hunan. These occurrence records originate from both cropland and natural habitats. Using ArcGIS (version 10.4; ESRI, Redlands, USA) software, the study screened species distribution sites and excluded occurrence records with a straight‐line distance of less than 5000 m to minimize sampling deviation (Yang, He, et al. 2023). Ultimately, 76 valid occurrence records were retained for analysis (Figure 1a, Table S1). Between October and December 2022, 10 samples from various origins were collected (Table 1, Figure 1b). Samples S1–S2, S3–S7, and S8–S10 were collected from low‐, medium‐, and high‐suitability habitats, respectively. Associate Professor Dexin Kong (South China Agricultural University) identified the samples as the fruit of *S. grosvenorii* (Figure 1c).

**TABLE 1:** *Siraitia grosvenorii* collection sites.

Sample no.	Location	Longitude	Latitude	Medicinal parts	Gatherer	Voucher specimen
S1	Qiangong Ping Town, Fenghuang County, Hunan	109°51′ E	28°03′ N	Fruit	Y. Yang	SG202201
S2	Dejiang County, Tongren, Guizhou	108°12′ E	28°26′ N	Fruit	T. Chen	SG202202
S3	Wuhua County, Meizhou, Guangdong	115°76′ E	23°93′ N	Fruit	L. Yang	SG202203
S4	Yanshan County, Zhuang and Miao Autonomous Prefecture, Wenshan, Yunnan	104°34′ E	23°6′ N	Fruit	Q. Yang	SG202204
S5	Ma Shan county, Nanning, Guangxi	108°18′ E	23°71′ N	Fruit	L. Yang	SG202205
S6	Huajiang Township, Xing'an County, Guilin, Guangxi	110°48′ E	25°77′ N	Fruit	Y. Yang	SG202206
S7	Longsheng Rthnic Autonomous County, Guilin, Guangxi	110°02′ E	25°81′ N	Fruit	Y. Yang	SG202207
S8	Guchen Village, Pingnan County, Guigang, Guangxi	110°45′ E	23°12′ N	Fruit	Y. Yang	SG202208
S9	Yongfu County, Guilin, Guangxi	110°02′ E	24°98′ N	Fruit	Y. Yang	SG202209
S10	Xiangzhou County, Laibing, Guangxi	109°7′ E	23°97′ N	Fruit	Y. Yang	SG202210

### 2.2 Environmental Variables

Nineteen bioclimatic variables (Table S2) were sourced from WorldClim (version 2.1, https://www.worldclim.org/). This research examines the current climate period (1970–2000), as well as future climate periods 2050S (2041–2060) and 2090S (2081–2100). Future climate data were derived from the Beijing Climate Center Climate System Model (BCC‐CSM) of the Coupled Model Intercomparison Project's sixth phase (CMIP6) and included two shared socioeconomic pathways (SSP126 and SSP585). BCC‐CSM is favored over other GCMs due to its superior performance (Zhan et al. 2022; Yang, He, et al. 2023) and is commonly used in China for assessing species distribution and evaluating the impact of future climate change on biodiversity (Liu et al. 2021; Zhang et al. 2021; Zhan et al. 2022; Yang, He, et al. 2023). SSP126 and SSP585 represent the most optimistic and pessimistic scenarios of future greenhouse gas emissions, with radiation intensities of 2.6 and 8.5 W·m^−2^ respectively (Zhan et al. 2022; Yang, He, et al. 2023). Additionally, 15 soil variables and 3 topographic variables (Table S2) were obtained from the Harmonized World Soil Database (version 1.2, https://www.fao.org/soils‐portal/en/) and WorldClim. All 37 of the downloaded environmental factors have a spatial resolution of 2.5 min.

Correlations and multicollinearity among environmental variables can cause overfitting and misinterpretation, affecting the accuracy of the MaxEnt model and leading to unrealistic suitable habitat areas (Gillett et al. 2016; Li, Fan, and He 2020; Zhan et al. 2022; Yang, He, et al. 2023). Therefore, Spearman's correlation analysis was conducted on the values obtained by coupling environmental variables with 76 samples using SPSS (version 20.0; IBM, New York, USA). During the simulation, we ranked all 37 environmental factors by importance and, for any set whose pairwise correlation exceeded 0.7, retained only the single most important factor (Figure S1) (Ranjitkar et al. 2014; Zhan et al. 2022; Yang, He, et al. 2023). Consequently, six climate variables: annual mean temperature (Bio_1), mean diurnal range (Bio_2), temperature seasonality (Bio_4), precipitation seasonality (Bio_15), precipitation of wettest quarter (Bio_16), and precipitation of warmest quarter (Bio_18); six soil variables: soil available water content (Awc_class), substratesoil clay content (S_clay), substratesoil organic carbon (S_oc), topsoil carbonate or lime content (T_caco3), topsoil organic carbon (T_oc), and topsoil sand content (T_sand); three topographic variables: aspect, elevation (Elev), and slope were selected for distribution prediction.

### 2.3 Species Distribution Modeling

The MaxEnt model was employed to predict, analyze, and evaluate potentially suitable habitats for *S. grosvenorii* under various climate scenarios. Input data included the occurrence records of *S. grosvenorii* and relevant environmental variables. The model used 25% of the data for performance assessment, with the remaining 75% allocated for training. Cross‐validation was performed 10 times with a maximum of 10^6^ iterations (Zhao et al. 2021; Zhan et al. 2022). The Jackknife test and response curves were used to evaluate the contributions and thresholds of environmental variables to potentially suitable habitats for *S. grosvenorii* in China. MESS analysis was conducted to enhance the reliability of model projections under future climate scenarios. The area under the receiver operating characteristic (ROC) curve (AUC) measured the accuracy of the MaxEnt model predictions (Zhan et al. 2022; Yang, He, et al. 2023).

Post‐modeling, the results of the probability of presence were classified using ArcGIS. Suitable areas for *S. grosvenorii* were categorized based on the Maximum Test Sensitivity Plus Specificity (MTSPS) and natural breaks method criterion (Ramos et al. 2019; Yang, He, et al. 2023) (Table S3) into four categories: unsuitable habitats (*p* < MTSPS), low‐suitable habitats (MTSPS ≤ *p* < 0.5), medium‐suitable habitats (0.5 ≤ *p* < 0.7), and high‐suitable habitats (*p* ≥ 0.7).

### 2.4 Centroid Migration in the Core Distribution

The SDM tool in ArcGIS is a widely used package for calculating centroids of suitable areas for *S. grosvenorii* across two time periods (current and future) and various climate change scenarios. It also determines the species' migration direction over time and calculates the distance of centroid migration (Brown et al. 2017). The methodology involves first converting the simulation's prediction results of potentially suitable areas into vector binary format, where species suitability probability of *p* ≥ MTSPS is considered suitable, and *p* < MTSPS is considered non‐suitable. Using the Zonal Geometry feature within the Spatial Analysis Tools, centroid coordinates for *S. grosvenorii* were identified based on different climate projections. Suitability areas for future climates were then categorized into four types: no change (areas that remain suitable in both future and current climates), contraction (areas that will become unsuitable in the future), expansion (areas that will become newly suitable in the future), and unsuitable habitats (areas unsuitable in both future and current climates).

### 2.5 Samples Treatment and HPLC Analysis

Fresh mature fruit samples were randomly taken from three individual plants representing the local population for each specimen. After air drying, the samples were crushed into a 60‐mesh powder. Precisely 0.5 g of the powder was placed in a conical flask with a stopper, to which 50 mL of methanol was added. The flask was weighed and then refluxed for 2 h. After cooling, the flask was reweighed, and any weight loss was compensated with methanol before filtering. Twenty milliliters of the filtrate was taken, the solvent was evaporated to dryness, and 10 mL of water was added to dissolve the residue. The solution was passed through a large‐pore adsorption resin column AB‐8, eluted with 100 mL of water (discarding the aqueous solution), followed by 100 mL of 20% ethanol (discarding the eluate), and finally 100 mL of 70% ethanol. The final eluate was collected, and the solvent was evaporated to dryness. The residue was dissolved in a 23% acetonitrile aqueous solution and diluted to 10 mL (China Pharmacopoeia Commission 2025).

The MV content of the samples was assessed using HPLC (1260A, Agilent). The chromatographic column used was Agilent ZORBAX Eclipse Plus C 18 (4.6 mm × 250 mm, 5 μm). Elution was performed with a solvent mixture of 23% acetonitrile (solvent A) and 77% water (solvent B). The detection wavelength was set at 203 nm, with a column temperature of 30°C, an injection volume of 10 μL, and a flow rate of 1.00 mL·min^−1^. According to the PPRC, the MV content served as a quality evaluation standard to investigate the potential impact of habitat suitability on MV accumulation. The MV (CAS:88901‐36‐4; SYBT, Shanghai, China) standard curve was established by plotting five different peak areas (*x*, mAU) versus concentration (*y*, mg·mL^−1^) (*y* = 0.0005*x* − 0.0039, *r* ^*2*^ = 1) (Figure S2a).

### 2.6 Measurement of TFC and TPA

The extracting solution (500 μL) of the sample was evenly mixed with 5% sodium nitrite (30 μL) and allowed to react for 6 min. Subsequently, 10% aluminum nitrate (30 μL) was added, and the reaction was continued for another 6 min. Following this, a 4% sodium hydroxide solution (400 μL) was added, and the volume was adjusted to 1 mL with 70% methanol (40 μL). The solution was left to stand for 10 min before measuring the absorbance at OD_510nm_ (He et al. 2024; Yang et al. 2024). The same procedure was used to establish the standard curve for rutin (*y* = 1.7876*x* + 0.0544; *r* ^*2*^ = 0.9951) (Figure S2b), where *y* represents absorbance (nm) and *x* represents the concentration of rutin (mg·mL^−1^) (*n* = 3). TFC is expressed as rutin equivalents (mg TAE·g^−1^ DW).

For the TPC assay, 150 μL of the sample extraction solution was mixed with 10 μL of Folin–Ciocalteu reagent (1 mol·L^−1^) and allowed to stand for 6 min before adding 40 μL of 20% (w/v) sodium carbonate solution with thorough shaking. The mixture was incubated at 25°C for 1 h, and absorbance was measured at OD_765nm_ (He et al. 2024; Yang et al. 2024). The standard curve for gallic acid was established similarly (*y* = 14.072*x* + 0.0527; *r* ^*2*^ = 0.9959) (Figure S2c), where *y* represents absorbance (nm) and *x* represents the concentration of gallic acid (mg·mL^−1^) (*n* = 3). TPC is expressed as gallic acid equivalents (mg TAE·g^−1^ DW).

### 2.7 Antioxidant Analysis In Vitro

The ferric reducing/antioxidant power (FRAP) detection method kit (Beyotime Institute of Biotechnology, Shanghai, China) was utilized to assess antioxidant capacity. Antioxidant‐reducing agents in the samples reduce ferric‐tripyridyltriazine (Fe^3+^‐TPTZ) to form the blue complex Fe^2+^‐TPTZ. For each assay, 180 μL of FRAP working solution was added to a 96‐well plate, followed by 5 μL of each sample's extraction solution or Trolox (used as a positive control). The mixture was gently mixed and incubated at 37°C for 5 min. Absorbance was measured at OD_593nm_ using a multifunctional microplate detector (Infinite 200PRO). The antioxidant capacity of the samples was determined using the FeSO₄·7H₂O standard curve (*y* = 0.3825*x* + 0.0098; *r* ^*2*^ = 0.9987) (Figure S2d).

The 2,2′‐azino‐bis[3‐ethylbenzthiazoline‐6‐sulfonic acid] (ABTS^+^) radical cation decolorization analysis was conducted using a commercial kit following the manufacturer's instructions (Beyotime Institute of Biotechnology, Shanghai, China). Each assay involved adding 20 μL of peroxidase working solution to a 96‐well plate; then 10 μL of each sample was added and mixed thoroughly. Subsequently, 170 μL of ABTS working solution was added and mixed well, and the mixture was allowed to stand at room temperature for 6 min. Absorbance was measured at OD_734nm_. The antioxidant capacity of the samples was calculated using the Trolox standard curve (*y* = 0.9817*x* − 0.0902; *r* ^*2*^ = 0.9984) (Figure S2e). Trolox, a water‐soluble analog of vitamin E, was used as a reference standard to plot the calibration curve.

## 3 Results and Analysis

### 3.1 The Model Evaluation

The AUC value, ranging from 0 to 1, measures the prediction accuracy of the Maxent model. AUC values between 0.9 and 1 indicate high accuracy (Allouche et al. 2006; Zhao et al. 2021; Yang, He, et al. 2023). In this study, average AUC values ranged from 0.993 to 0.994 (Figure S3), demonstrating the exceptional accuracy and reliability of the Maxent model in calculating the environmental suitability for *S. grosvenorii* growth under various climatic scenarios.

### 3.2 Main Environmental Variables Affecting the Potential Distribution of *S. grosvenorii*

**FIGURE 2:**
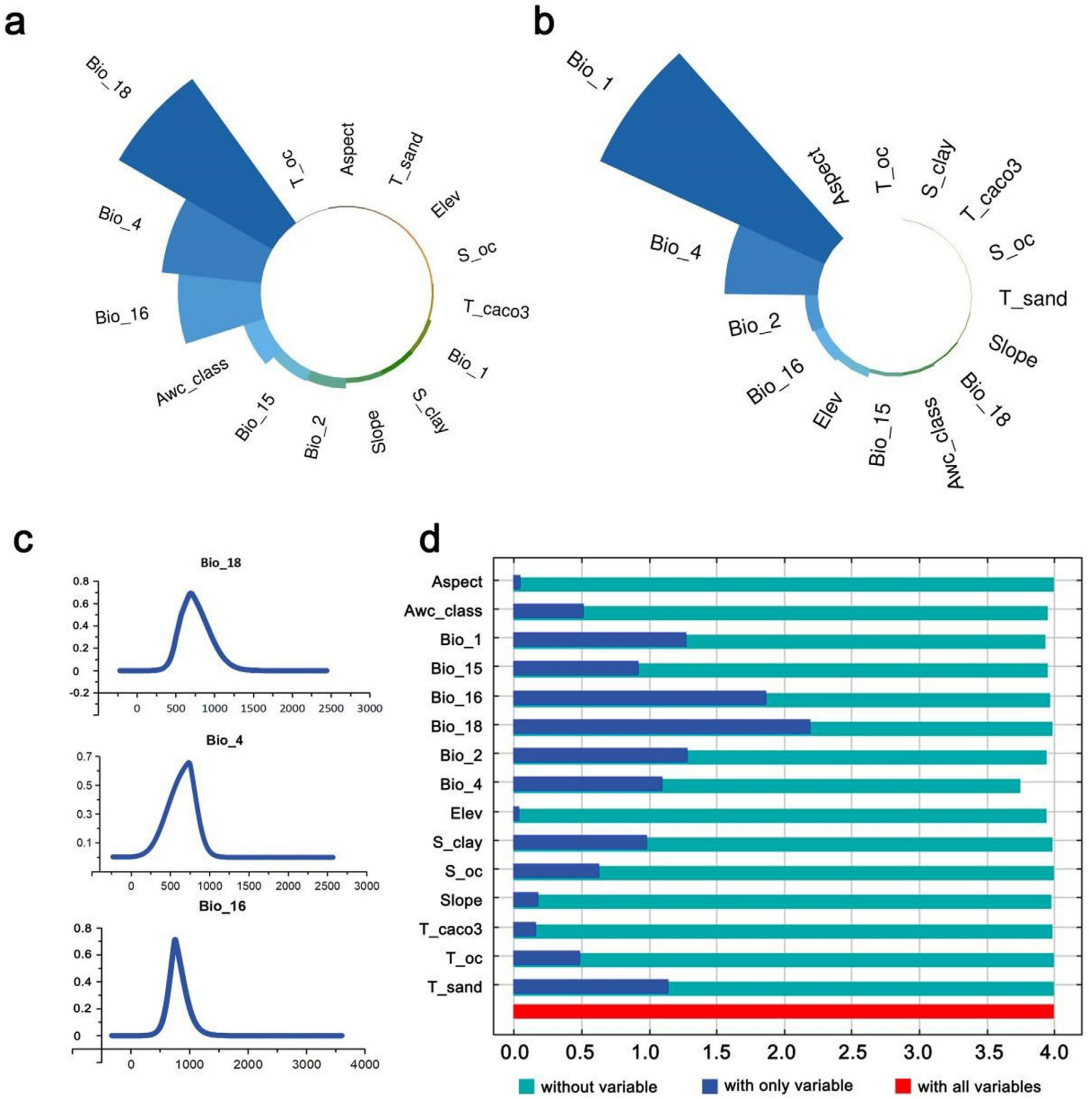
(a) Percentage contribution and (b) permutation importance of environmental variables under current climate scenarios. (c) Response curves of three bioclimatic variables. (d) Jackknife test of regularized training gain for *Siraitia grosvenorii*.

Figure 2a clearly illustrates that Bio_18, Bio_4, and Bio_16 are the primary variables influencing the distribution of *S. grosvenorii* under current climate scenarios, with percent contributions of 41.6%, 23.4%, and 19.4%, respectively. These variables cumulatively account for 84.4% of the total contribution. The response curves for these three bioclimatic variables exhibit a single‐peaked, normal distribution (Figure 2c). Bio_1 holds the highest permutation importance at 64.1%, followed by Bio_4 at 24.3% (Figure 2b). Bio_18, Bio_4, and Bio_16 also remain the top three variables affecting *S. grosvenorii* distribution under various future climate scenarios (Figure S4).

The Jackknife test identifies Bio_18, Bio_16, Bio_2, Bio_1, and T_sand as the top five environmental variables in single variable analysis across different climate scenarios (Figure 2d). Bio_18 has the highest weight, indicating it provides the most useful information independently. The absence of Bio_4 results in the greatest reduction in model gain, suggesting that Bio_4 contains the most unique information not present in other variables. Overall, precipitation factors (Bio_18, Bio_16, Awc_class, and Bio_15) and temperature factors (Bio_4, Bio_2, and Bio_1) are the main environmental determinants of the potential distribution of *S. grosvenorii*.

### 3.3 Distribution Prediction Under Current Climate Scenarios

**FIGURE 3:**
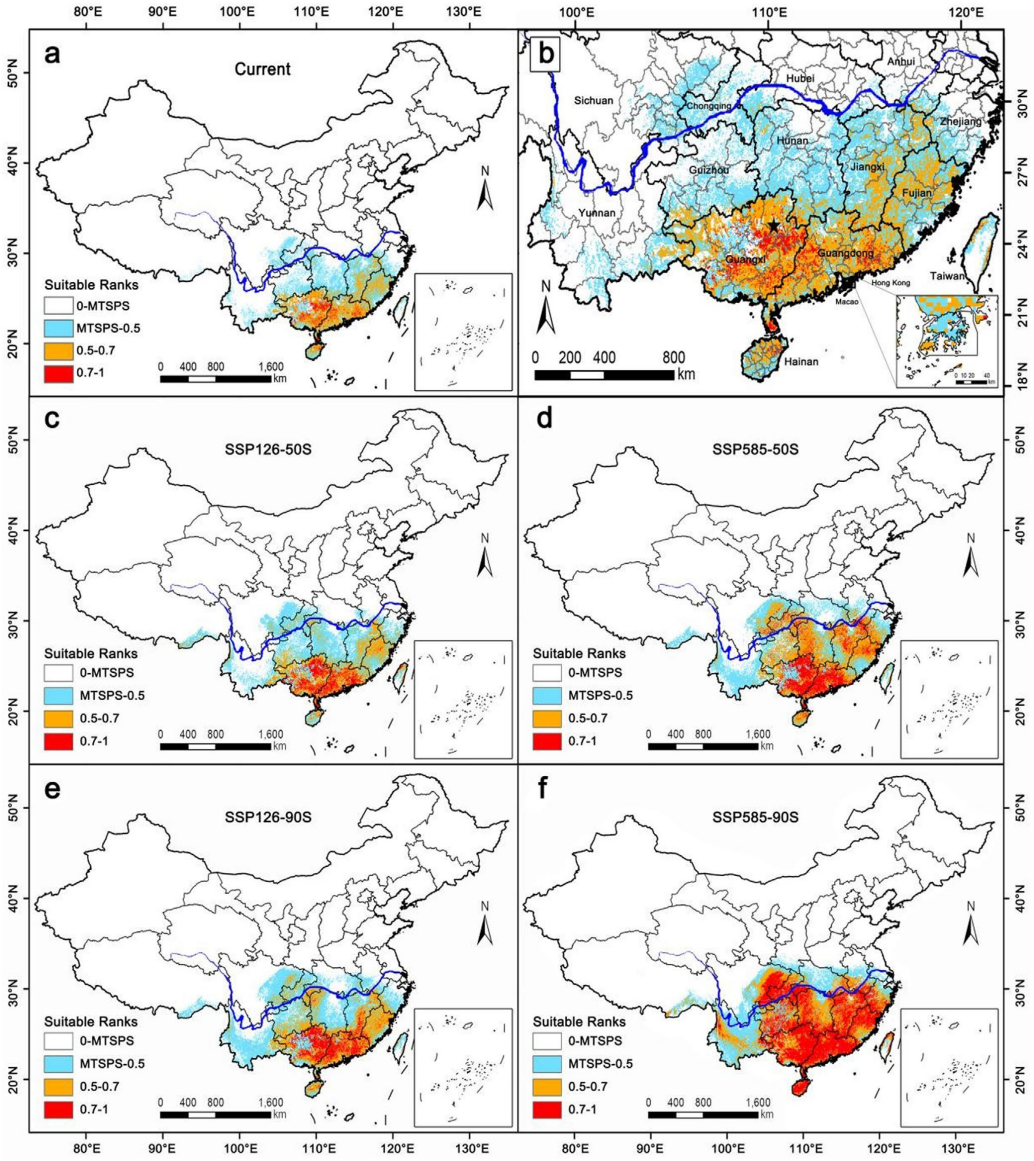
Distribution of suitable habitats for *Siraitia grosvenorii* in China under different climate scenarios. (a, b) Average for current; (c) Average for 2041–2060 (2050S), SSP126; (d) Average for 2041–2060 (2050S), SSP585; (e) Average for 2081–2100 (2090S), SSP126; (f) Average for 2081–2100 (2090S), SSP585.

The suitable habitat areas for *S. grosvenorii* were primarily distributed south of the Yangtze River (Figure 3a), particularly between latitudes 19° and 30° N, with a concentration between 22° and 26° N (Figure 3b). The total suitable habitat area (combining high‐ and medium‐suitability areas) for *S. grosvenorii* cultivation was 4,048,559.78 km^2^ (Table S4). High‐suitability habitats cover 533,261.66 km^2^, predominantly in Guangxi and Guangdong provinces. The largest distribution area was at the intersection of Guilin, Liuzhou, Wuzhou, Hezhou, and Laibin in Guangxi (Figure 3b, Star), which was currently the main production area for *S. grosvenorii* in China. This indicated that the modern potential distribution area simulated by MaxEnt closely matches current distribution records. Additional high‐suitability areas include Nanning, Yulin in Guangxi Province and Zhaoqing, Yunfu, Zhanjiang, Shanwei, Jieyang, Meizhou, and Heyuan in Guangdong Province. Medium‐suitability habitats, covering 3,515,298.12 km^2^, were primarily located in Guangxi, Guangdong, Hainan, Fujian, Jiangxi, and small parts of Hunan, Guizhou, Yunnan, Taiwan, Zhejiang, and Anhui provinces (Table S4). Low‐suitability habitats, totaling 5,814,630.65 km^2^, were scattered across Hunan, Jiangxi, Guizhou, Fujian, and the southern regions of Zhejiang, Hubei, Chongqing, Sichuan, and Yunnan provinces.

### 3.4 Distribution Prediction Under Future Climate Scenarios

Compared with Figure 3a,c–f clearly demonstrates a northward shift in total suitable habitat areas, particularly the high‐suitability areas, from coastal provinces to the Yangtze River Basin and Sichuan Basin (southeast Sichuan and west Chongqing) in the future. Under the emission scenarios SSP126‐2050S, SSP585‐2050S, SSP126‐2090S, and SSP585‐2090S, the total suitable habitat areas for *S. grosvenorii* were projected to be 5,335,841.96, 9,456,972.63, 6,718,989.38, and 14,137,347.13 km^2^, respectively, representing increases of 31.80%, 133.59%, 65.96%, and 249.19% compared to current climate scenarios (Table 2). High‐suitability habitats were expected to increase by 181.45%, 489.31%, 271.51%, and 1451.08%, while medium‐suitability habitats were projected to increase by 9.09%, 79.63%, 34.78%, and 66.87%, respectively, compared to current conditions.

**TABLE 2:** Area and increase rate of suitable habitat for *Siraitia grosvenorii* under different future climate scenarios.

Periods	Climate scenarios	Low‐suitable habitats		Medium‐suitable habitats		High‐suitable habitats		Total suitable habitats	
		Area (km^2^)	Rise rate	Area (km^2^)	Rise rate	Area (km^2^)	Rise rate	Area (km^2^)	Rise rate
2041–2060	SSP126	7,655,243.47	31.65%	3,834,968.42	9.09%	1,500,873.54	181.45%	5,335,841.96	31.80%
	SSP585	6,936,343.68	19.29%	6,314,384.27	79.63%	3,142,588.36	489.31%	9,456,972.63	133.59%
2081–2100	SSP126	8,655,467.46	48.86%	4,737,893.65	34.78%	1,981,095.73	271.51%	6,718,989.38	65.96%
	SSP585	6,187,698.79	6.42%	5,866,057.43	66.87%	8,271,289.71	1451.08%	14,137,347.13	249.19%

Under SSP126 scenarios, the total suitable habitat areas, including high‐ and medium‐suitability areas, were projected to increase, but high‐suitability habitats will remain primarily in Guangxi and Guangdong provinces over the next 80 years. Conversely, under SSP585 scenarios, high‐suitability habitats were anticipated to rapidly expand northward, including the Sichuan Basin and nearly all provinces south of the Yangtze River, except Yunnan. Overall, future climate scenarios indicate a northward shift in total suitable habitat areas for *S. grosvenorii*, with a significant expansion in high‐suitability areas.

### 3.5 Changes in the Distribution Core Under Different Climate Scenarios

**FIGURE 4:**
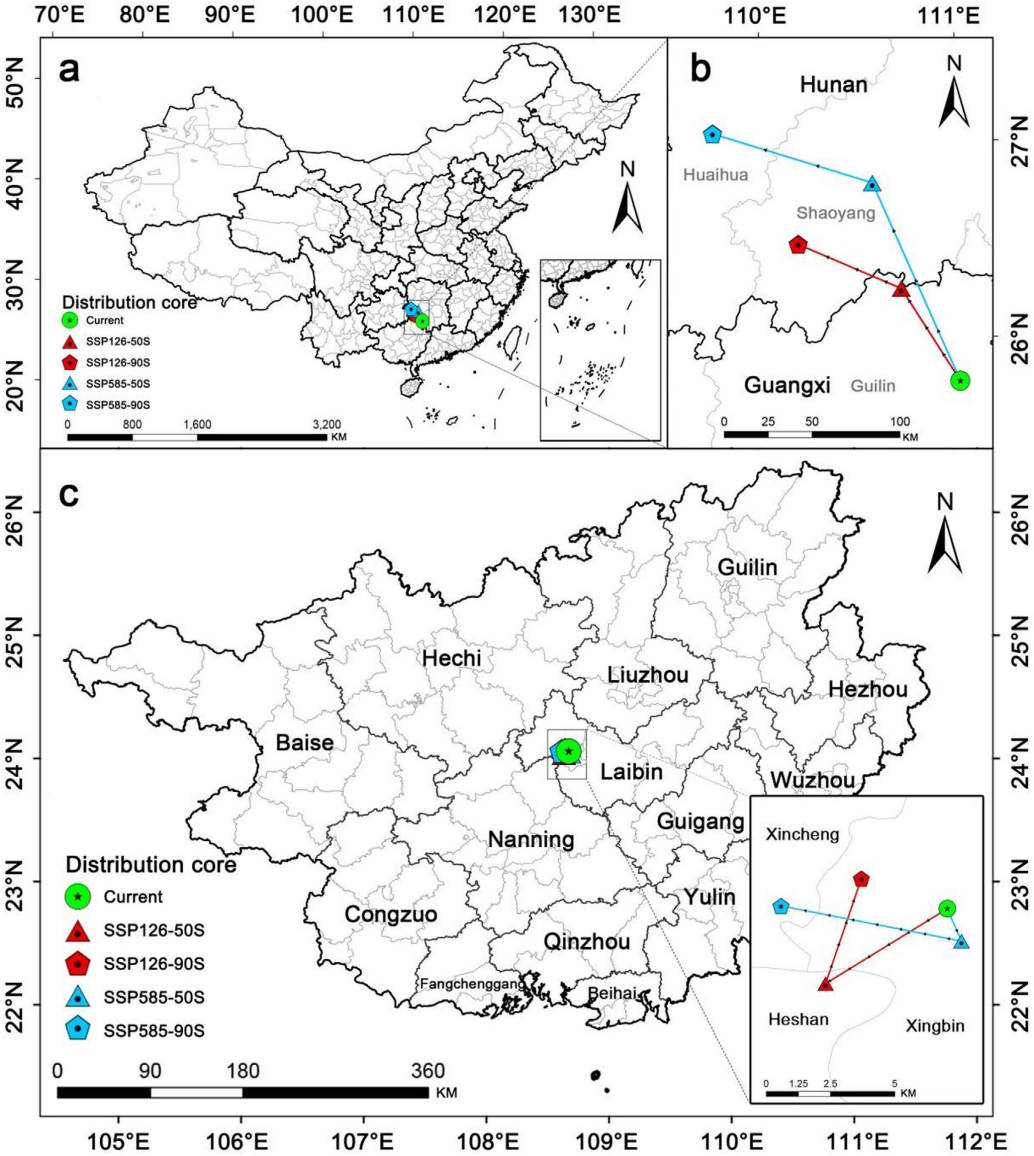
Migration routes of distribution cores of *Siraitia grosvenorii* under different climate scenarios in China (a, b) and Guangxi Province (c).

The distribution cores of *S. grosvenorii* in China and Guangxi Province shift under different future climate scenarios (Figure 4). Currently, the core distribution in China is centered in Guilin City, Guangxi Province (111.03° E, 25.77° N; Figure 4a,b). Future climate scenarios predict a northwestward shift for all distribution cores (Figure 4b). Under SSP126, the core in the 2050s is expected to move to 110.73° E, 26.24° N in the same city (a migration distance of 60.45 km), and to Shaoyang City, Hunan Province (110.20° E, 26.47° N; a migration distance of 58.22 km) in the 2090s. Similarly, under SSP585, the core is predicted to shift to Shaoyang City, Hunan Province (110.58° E, 26.78° N; a migration distance of 89.45 km) in the 2050s, and to Huaihua City, Hunan Province (109.77° E, 27.03° N; a migration distance of 161.90 km) in the 2090s.

The current core of suitable habitat in Guangxi Province is located in Xingbin District, Laibin City (108.89° E, 23.84° N; Figure 4c). Under SSP126, the core is projected to move southwest to Heshan County, Laibin City (108.84° E, 23.82° N; a migration distance of 5.22 km) in the 2050s, and to the northeast to Xingbin District (108.86° E, 23.85° N; a migration distance of 4.28 km) in the 2090s. Similarly, under SSP585 in the 2050s and 2090s, the cores are predicted to shift to Xingbin District (108.89° E, 23.83° N; a migration distance of 1.36 km) and Xincheng County, Laibin City (108.83° E, 23.84° N; a migration distance of 6.57 km), respectively.

### 3.6 Quantitation of MV in *S. grosvenorii* by HPLC

**FIGURE 5:**
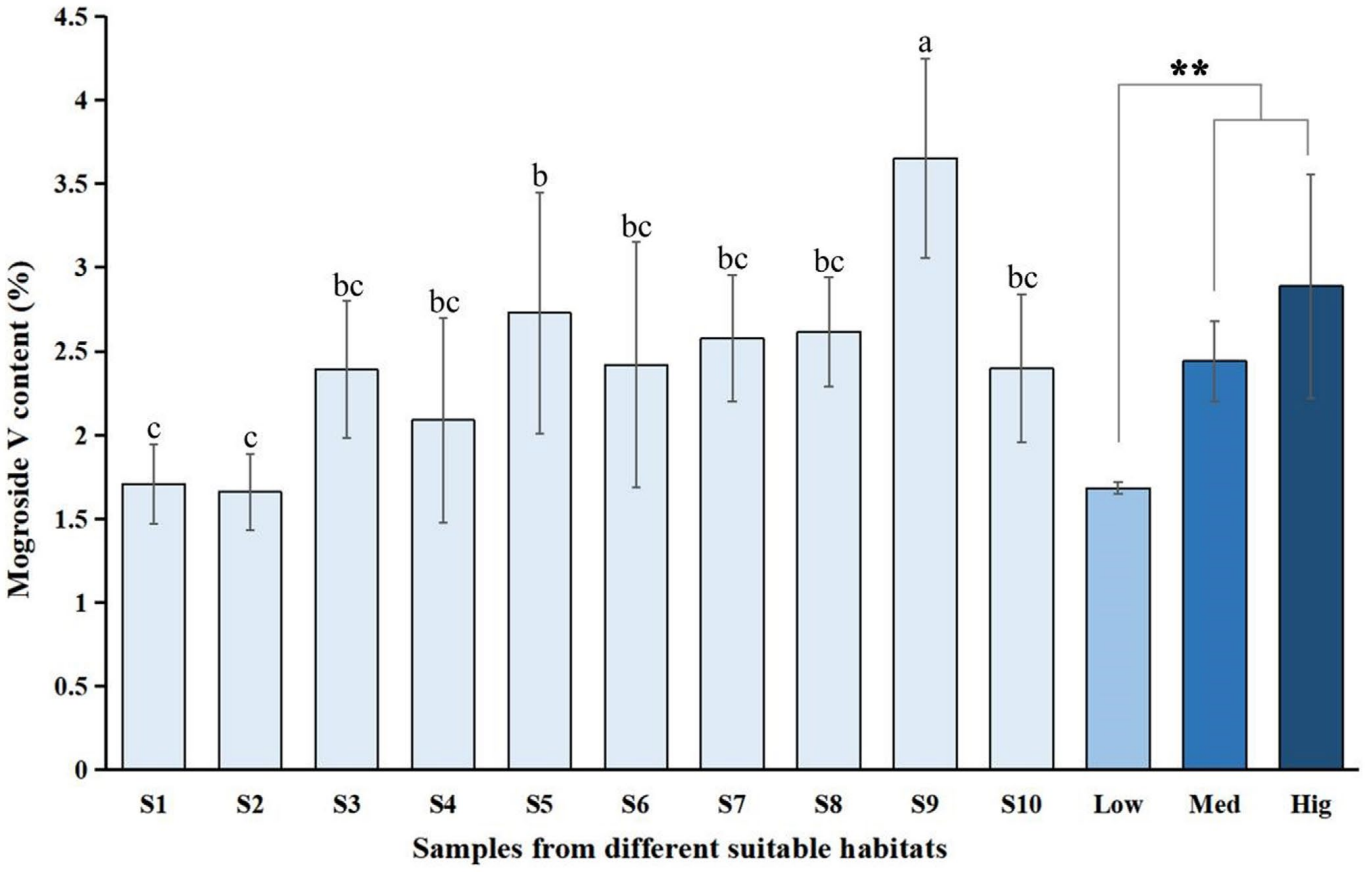
Mogroside V content of *Siraitia grosvenorii* from different suitable habitats. S1–S10 represent samples 1 to 10 from various suitable habitats. “Low” denotes the average mogroside V content of S1–S2 (low‐suitable habitats). “Med” denotes the average mogroside V content of S3–S7 (medium‐suitable habitats). “Hig” denotes the average mogroside V content of S8–S10 (high‐suitable habitats). Different letters (“a, b, c”) and “**” indicate significant differences among samples (*p* < 0.05).

The HPLC results indicated that the MV content in all samples (S1–S10) met the quality evaluation standard of medicinal materials (0.5%) stipulated by the PPRC. However, the MV content varied among the 10 samples from different suitable habitats (Figure 5). The highest MV content was observed in S9 (3.65%) from Yongfu County, Guilin City, Guangxi Province, significantly surpassing other samples (*p* < 0.05). This was followed by S5 from Masan County, Nanning City, Guangxi Province (2.73%), and S8 from Guchen Village, Pingnan County, Guigang City, Guangxi Province (2.62%). Samples with MV content below 2.00% included S2 (1.66%) from Guizhou Province and S1 (1.71%) from Hunan Province. Samples S1–S2, S3–S7, and S8–S10 originated from low‐, medium‐, and high‐suitability habitats, respectively. The average MV content of *S. grosvenorii* grown in high‐ and medium‐suitability habitats was significantly higher (*p* < 0.05) than that in low‐suitability habitats, indicating that the quality of herbal medicine cultivated in high‐ and medium‐suitability habitats is superior to that from low‐suitability habitats.

### 3.7 Antioxidant Properties

As shown in Table 3, S2, S1, and S4 ranked highest in TFC and TPA content. The top three samples with the highest antioxidant activity in the ABTS assay were S1, S2, and S5, while S2, S1, and S4 exhibited the highest antioxidant activity in the FRAP assay. Although minor variations were observed between the two assays, samples S1 and S2 consistently showed strong antioxidant activity. The antioxidant potency composite index (APCI) was employed to determine the antioxidant capacity of the 10 samples (He et al. 2024; Yang et al. 2025). The results indicated that S2 had the highest comprehensive antioxidant index, followed by S1 and S4, corroborating the high concentrations of TFC and TPA in these samples. Samples from low‐suitability habitats displayed significantly higher mean TFC and TPA content compared to those from medium‐ and high‐suitability habitats (*p* < 0.05). Similarly, the in vitro APCI of samples from low‐suitability habitats was markedly superior to those from medium‐ and high‐suitability habitats.

**TABLE 3:** Total flavonoid content (TFC), total phenolic acid (TPA), and in vitro antioxidant activity of *Siraitia grosvenorii* from different habitats.

Sample	TFC (mg g^−1^)	TPA (mg g^−1^)	ABTS (mg TAE·g^−1^ DW)	FRAP (mg TAE·g^−1^ DW)	APCI (%)	Sort
S1	3.87 ± 0.21^ab^	1.66 ± 0.06^b^	17.47 ± 0.13^a^	18.42 ± 0.56^a^	98.37	2
S2	4.15 ± 0.44^a^	2.20 ± 0.05^a^	17.10 ± 0.17^b^	19.04 ± 0.68^a^	98.93	1
S3	3.01 ± 0.34^cd^	1.14 ± 0.02^e^	16.02 ± 0.09^cd^	13.05 ± 0.63^d^	80.11	5
S4	3.86 ± 0.63^ab^	1.57 ± 0.07^c^	15.48 ± 0.19^e^	16.38 ± 0.85^b^	87.34	3
S5	2.54 ± 0.19^de^	1.37 ± 0.02^d^	16.29 ± 0.15^c^	14.37 ± 0.38^c^	84.37	4
S6	3.31 ± 0.18^bc^	1.31 ± 0.02^d^	15.91 ± 0.18^d^	11.15 ± 0.24^e^	74.80	7
S7	3.45 ± 0.54^abc^	1.11 ± 0.03^e^	15.48 ± 0.23^e^	12.80 ± 0.34^d^	77.91	6
S8	2.30 ± 0.16^e^	0.94 ± 0.02^f^	14.44 ± 0.29^f^	10.30 ± 0.20^ef^	68.36	8
S9	1.42 ± 0.35^f^	0.13 ± 0.01^g^	10.76 ± 0.20^h^	9.94 ± 0.42^f^	56.90	10
S10	2.09 ± 0.48^e^	0.97 ± 0.04^f^	11.27 ± 0.36^g^	10.66 ± 0.73^ef^	60.27	9
LES	4.01 ± 0.34^A^	1.93 ± 0.30^A^	17.28 ± 0.25^A^	18.73 ± 0.65^A^	98.65	R1
MOD	3.23 ± 0.58^B^	1.30 ± 0.18^B^	15.84 ± 0.36^B^	13.55 ± 1.87^B^	80.91	R2
HIG	1.94 ± 0.50^C^	0.59 ± 0.55^C^	12.16 ± 1.74^C^	10.30 ± 0.53^C^	61.84	R3

*Note:* LES was the mogroside V average content of S1–S2. MOD was the mogroside V average content of S3–S7. HIG was the mogroside V average content of S8–S10. Different letters (“a, b, c, d, e, f, g, h” and “A, B, C”) indicate significant differences among samples (*p* < 0.05).

Abbreviations: ABTS, 2,2′‐azino‐bis[3‐ethlbenzthiazoline‐6‐sulfonic acid]; APCI, antioxidant Potency Composite Index; FRAP, ferric reducing/antioxidant power.

## 4 Discussion

### 4.1 The Dominant Environmental Factor Affecting the Distribution of *S. grosvenorii*

**FIGURE 6:**
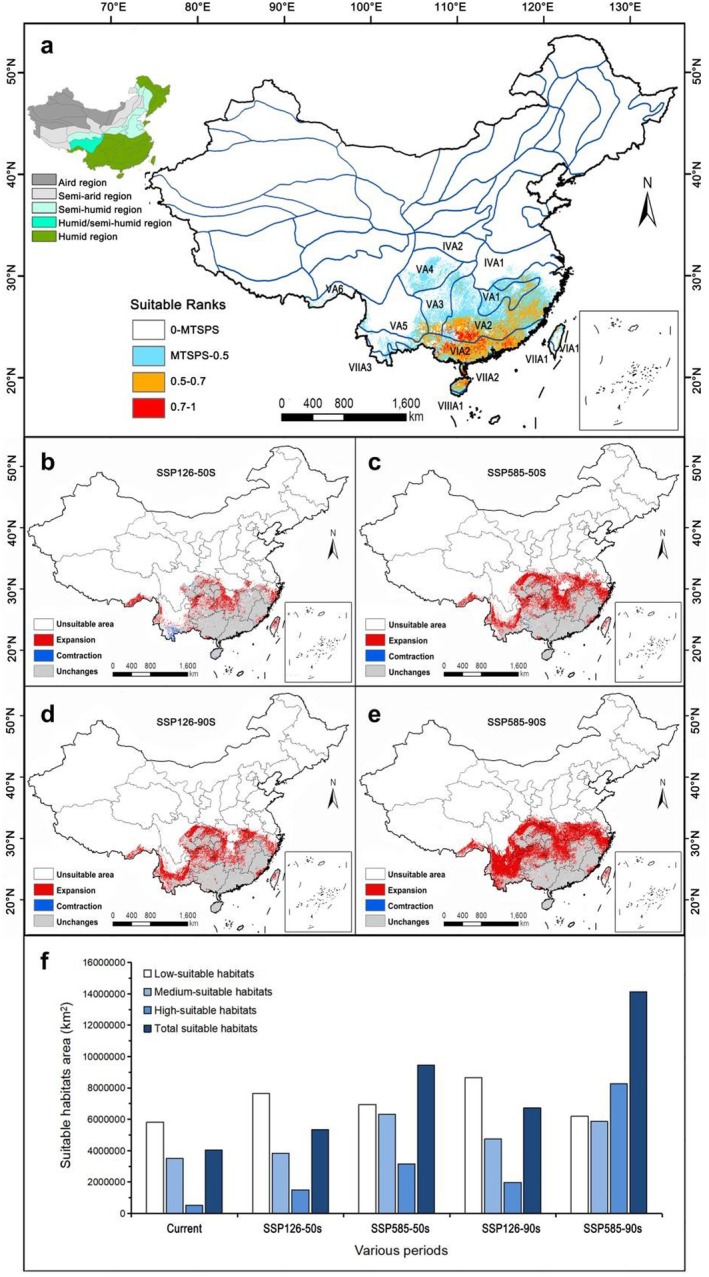
(a) The distribution of suitable habitat area of *Siraitia grosvenorii* in the contemporary climate environment. (b–e) Spatial changes of suitable habitat area of *S*. *grosvenorii* in China under different climate scenarios. (f) The suitable habitat areas (km^2^) of *S*. *grosvenorii* under different climate scenarios.

The comprehensive impact of environmental variables is a primary driving factor in the spatial distribution patterns of plants, with significant differences in the dominant factors limiting the distribution of various species. Water and thermal conditions often play a leading role (Xie et al. 2024). Previous research has identified precipitation and temperature as the main environmental factors affecting the distribution of medicinal materials such as *Zanthoxylum nitidum* and *Panax notoginseng* (Zhan et al. 2022; Yang, He, et al. 2023). Wei et al. (2020) used the GMPGIS system to analyze the ecological factors influencing *S. grosvenorii* globally, finding that temperature, sunlight, and precipitation are the primary factors, aligning with the findings of this study. Figure 6a shows that the current distribution of *S. grosvenorii* is located in the humid regions of China, primarily influenced by the summer monsoon, which provides abundant precipitation essential for the plant's growth. This observation is consistent with previous research (Editorial Committee of Flora of China, Chinese Academy of Sciences 2007). As a climbing herbaceous plant, *S. grosvenorii*, categorized as shallow‐rooted, requires timely irrigation during the summer to enhance survival and yield in field cultivation (Editorial Committee of Flora of China, Chinese Academy of Sciences 2007; Yang 2020; Yang 2021). Therefore, precipitation is indeed the most crucial factor affecting the distribution of *S. grosvenorii*, with precipitation‐related factors (Bio_18 and Bio_16) accounting for 61.0% of the contribution percentage.

Plants require an optimal temperature range for normal growth, with photosynthesis and respiration occurring within specific temperature thresholds (Ma et al. 2010). Low temperatures can cause frost and freeze damage, severely inhibiting plant growth (Ladwig et al. 2016). The optimal average annual growth temperature for *S. grosvenorii* is 19°C–22°C. This plant is native to the Longji Mountain area of Guilin City, Guangxi Province, China, and is uniquely adapted to the southern regions of China (Editorial Committee of Flora of China, Chinese Academy of Sciences 2007). Research indicates that *S. grosvenorii* is predominantly distributed in tropical and subtropical monsoon climate zones, particularly in the southern subtropical (IVA2), central subtropical (VA2), and northern subtropical (VIIA2) regions (Figure 6a). Temperature is another critical factor influencing the distribution of *S. grosvenorii* in China, with Bio_4 and Bio_1 contributing 24.4% to the distribution model and permutation importance of 88.4%. Additionally, factors such as Awc_class, Slope, Elev, and Aspect indirectly affect temperature and precipitation, altering the species' distribution pattern (Wang et al. 2023; Xie et al. 2024). The remaining environmental factors contribute less significantly and serve a compensatory role in the distribution of *S. grosvenorii*.

### 4.2 Analysis of the Spatial Distribution Pattern Changes in the Potential Suitable Habitats of *S. grosvenorii*

Assessing potential distribution changes in species under climate change is essential for evaluating impacts and developing conservation strategies to maintain ecological balance (Deb, Phinn, et al. 2017; Wang et al. 2023). Different species respond to climate change in varying degrees. This study employs the MaxEnt model and ArcGIS software to simulate the potential geographical distribution of *S. grosvenorii* over different periods, revealing a distribution range between 18°–35° N and 91°–122° E. These findings align with the field survey results of Li et al. (2007), indicating high simulation accuracy and effectiveness of the MaxEnt model for *S. grosvenorii*. Climate remains the most critical factor influencing species survival and distribution, with plant distribution characteristics being a direct response to climatic conditions (Duan et al. 2020). Geographical distribution maps show *S*. *grosvenorii* predominantly south of the Yangtze River, particularly in Guangxi Province, a highly suitable habitat for the species. This is consistent with previous research and distribution surveys (Editorial Committee of Flora of China, Chinese Academy of Sciences 2007; Wei et al. 2020).

Compared to the current climate scenario, future projections show an expansion of potentially suitable habitats for *S. grosvenorii* in varying degrees across four periods (Figure 6b–f), suggesting good adaptability to future climate changes. However, the SSP126‐50s scenario projects a slight reduction in suitable habitat in southern Yunnan Province (Figure 6b). The high‐concentration emission scenario (SSP585) for the 2050s and 2090s shows a greater expansion rate than the low‐concentration scenario (SSP126), with a significant northward shift to the Qinling‐Huaihe area (Figure 6b–e). The national distribution centroid of *S. grosvenorii* moves northward under all future climate scenarios (Figure 4b), confirming a potential northward shift in suitable habitats. This pattern mirrors findings in the study of *Z. nitidum* (Yang, He, et al. 2023), suggesting that high‐concentration emissions will likely increase temperatures and rainfall south of the Qinling‐Huaihe line. The results imply an optimistic future for *S. grosvenorii* habitats over the next 80 years but also highlight significant environmental changes, particularly in precipitation and temperature, already occurring or expected to alter the distribution areas of many medicinal plants in China (Wang et al. 2019; Guo et al. 2021; Shen et al. 2021; Zhan et al. 2022; Yang, He, et al. 2023).

### 4.3 The Correlation Between the Quality of *S. grosvenorii* and Its Suitable Habitat

**FIGURE 7:**
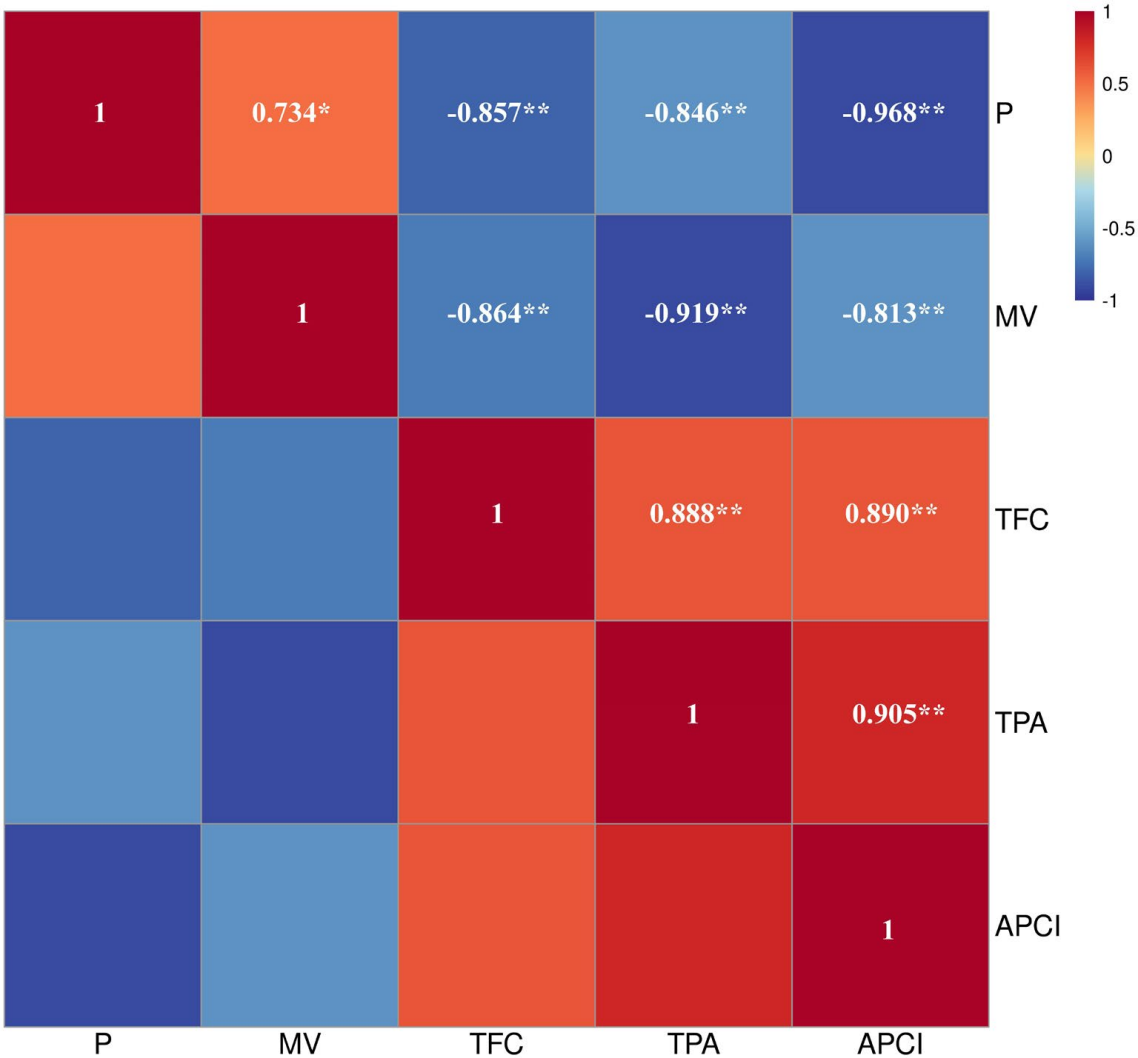
Correlation heatmap analysis between P and MV, TFC, TPA, APCI. APCI, antioxidant potency composite index; MV, mogroside V; P, predicted probability values; TFC, total flavonoid content; TPA, total phenolic acid. “*” indicates significant correlation at the 0.05 level and “**” indicates significant correlation at the 0.01 level.

Environmental factors are the primary constraints for the survival, growth, and secondary metabolite accumulation in medicinal plants (Wang et al. 2019; Li, Kong, Fu, et al. 2020; Pant et al. 2021). Variations in precipitation, temperature, light intensity, and salinity significantly impact the physiological traits and secondary metabolite levels in medicinal plants (Li, Kong, Fu, et al. 2020; Pant et al. 2021). Similar studies on medicinal plants like *Cistanche*, *Gentiana rigescens*, *Ophiocordyceps sinensis*, and *Z. nitidum* have demonstrated that bioactive component levels are higher in suitable habitats (Wang et al. 2019; Guo et al. 2021; Shen et al. 2021; Yang, Li, et al. 2023). MV, a unique bioactive component in *S. grosvenorii*, is a quality evaluation standard in the PPRC (China Pharmacopoeia Commission 2025). This study confirmed that all *S. grosvenorii* samples met pharmacopeia standards, and their predicted probability values (*p*) were positively correlated with the content of the active compound MV (Figure 7). Specifically, the MV content was significantly higher in high‐ and medium‐suitability habitats than in those of low‐suitability habitats. This finding aligns with Zhang et al. (2023), who evaluated *S. grosvenorii* from 15 production areas in China. Furthermore, Guangxi Province, the origin of *S. grosvenorii*, has been recognized for superior quality and grade for thousands of years (Editorial Committee of Flora of China, Chinese academy of sciences 2007; China Pharmacopoeia Commission 2025), supporting this study's accuracy. Thus, in terms of MV content, *S. grosvenorii* from suitable habitats (high‐ and medium‐suitability) demonstrates better herbal quality.

Assessing medicinal material quality based solely on a single component is insufficient and does not meet the modernization requirements of traditional Chinese medicine (Waris et al. 2022; Yang, Li, et al. 2023; Anmol et al. 2024). Over 100 compounds have been identified in *S. grosvenorii*, including 46 triterpenes, 19 amino acids, 7 flavonoids, and 2 polysaccharides (Guo et al. 2024; Huang et al. 2024). This study found that the predicted probability values (*p*) of *S. grosvenorii* were negatively correlated with TFC, TPA, and APCI. Specifically, samples from low‐suitability habitat exhibited significantly higher TFC and TPA contents than those from high‐ and medium‐suitability habitats, along with stronger in vitro antioxidant capacity. This may be due to plants in low‐suitability habitats, such as those experiencing drought, low temperature, and salinity, increasing the synthesis of secondary metabolites like flavonoids to protect against oxidative stress, UV damage, or other environmental pressures (Li, Kong, Fu, et al. 2020; Pant et al. 2021). Flavonoids provide antioxidant protection and maintain cell membrane integrity (Rodríguez et al. 2023; He et al. 2024); they modulate stress signaling by regulating pathways such as NF‐κB and Nrf2 and enhance plant immune responses (Patil et al. 2024; Chen et al. 2025). In addition, flavonoids offer protective effects against stresses including UV radiation, low temperature, and salinity–alkalinity (Laoué et al. 2022; Zhao et al. 2024). Therefore, regarding TFC, TPA, and in vitro antioxidant capacity, the quality of *S. grosvenorii* from low‐suitability habitats is superior.

### 4.4 Conservation and Development of *S. grosvenorii* Under the Background of Climate Change

Under future climate scenarios, the total suitable habitat area for *S. grosvenorii* exhibits an increasing trend, with the range expanding northward and filling in previously unsuitable areas in southern provinces. This expansion presents significant potential for production and application, as the newly suitable areas can be utilized for the introduction and cultivation of *S. grosvenorii*. Field cultivation has demonstrated that *S. grosvenorii* possesses a degree of environmental adaptability, having been successfully introduced and cultivated in Hunan, Guizhou, and Yunnan Provinces (Wei et al. 2020; Yang 2020, 2021). However, in regions north of Guangxi Province, such as Fenghuang County, the growth of *S. grosvenorii* is often hampered, and yield losses can occur, due to low temperatures from spring “late cold spells” and the hot, dry conditions of summer (Yang 2020, 2021). Based on the suitable habitats identified in this study, the distribution range of *S. grosvenorii* could potentially expand to provinces such as Hubei, Anhui, and Zhejiang, thereby enhancing its economic and ecological value. High‐suitability habitats should implement efficient management models to promote resource development and utilization, establishing these areas as primary production zones for *S. grosvenorii*. Given its status as a valuable medicinal and edible traditional Chinese medicine (TCM) material unique to China, it is recommended to prioritize the main concentrated areas of high‐suitability habitats for protection. Strengthening environmental monitoring and management in these areas will support the conservation of *S. grosvenorii* germplasm resources. As the distribution range of *S. grosvenorii* is expected to expand under different climate scenarios, adaptive management strategies should be employed to effectively address the impacts of climate change. This includes planning and implementing conservation measures that ensure the sustainable development of *S. grosvenorii* resources.

## 5 Conclusions

This study utilized the MaxEnt model to evaluate and predict the distribution of *S. grosvenorii* in China under various climate scenarios for the first time. To determine the relationship between habitat suitability and MV content, TFC, TPA, and APCI were analyzed. The study yielded the following key findings: (1) Precipitation and temperature were identified as the crucial factors influencing the distribution of *S. grosvenorii*, with Bio18, Bio4, and Bio16 being the top three key variables. (2) Currently, suitable habitats for *S. grosvenorii* in China are primarily located south of the Yangtze River, with high‐suitability areas in specific regions of Guangxi and Guangdong Provinces. Medium‐suitability habitats are mainly distributed in Guangxi, Guangdong, Hainan, Fujian, and Jiangxi Provinces. (3) In the future, the suitable habitat area for *S. grosvenorii* is expected to expand northward. (4) MV content in high‐ and medium‐suitability habitats is significantly higher than in low‐suitability habitats. However, (5) TFC, TPA, and APCI in low‐suitability habitats are significantly higher than in high‐ and medium‐suitability habitats. Overall, these findings provide critical insights for identifying suitable areas for *S. grosvenorii* cultivation and evaluating the quality of *S. grosvenorii* resources in China.

## Author Contributions

**Yang Yang:** conceptualization (lead), funding acquisition (lead), supervision (equal), writing – review and editing (equal). **Mingli Hu:** methodology (equal), writing – original draft (equal). **Leilei Yang:** software (lead), visualization (equal). **Jingyuan Wang:** data curation (equal), software (equal). **Tao Tian:** data curation (equal), formal analysis (equal). **Wenyang Qing:** investigation (equal). **Shengmei Yang:** resources (equal). **Jun He:** investigation (equal). **Qing Yang:** funding acquisition (equal), validation (equal), writing – original draft (equal). **Sisheng Zhang:** conceptualization (equal), supervision (equal), writing – review and editing (equal).

## Funding

This work was supported by the Doctoral Scientific Research Foundation of Hunan University of Medicine (Document number 202543); the Hunan Provincial Natural Science Foundation (grant number 2024JJ7330); the Excellent Youth Project of Hunan Provincial Department of Education (grant number 24B1083); and the Wenshan Prefecture Wang Zhimin Expert Workstation Project (grant number 2021‐2).

## Conflicts of Interest

The authors declare no conflicts of interest.

## Supporting information


**Appendix S1:** ece372600‐sup‐0001‐AppendixS1.docx.

## Data Availability

All of the distribution site data of *Siraitia grosvenorii* used in this study is available and is provided in the Supporting Information.
