# Supplementary material for: Assessment of Suitable Habitats and Quality of Siraitia grosvenorii in China
Source: Ecol Evol. 2025 Nov 29;15(12):e72600. doi: 10.1002/ece3.72600 (PMC12664284; doi:10.1002/ece3.72600)
Supplement: Supplementary file 1 — Appendix S1: ece372600‐sup‐0001‐AppendixS1.docx. [file ECE3-15-e72600-s001.docx]

**Assessment of suitable habitats and quality of *Siraitia grosvenorii* in China**

**Supplementary files**


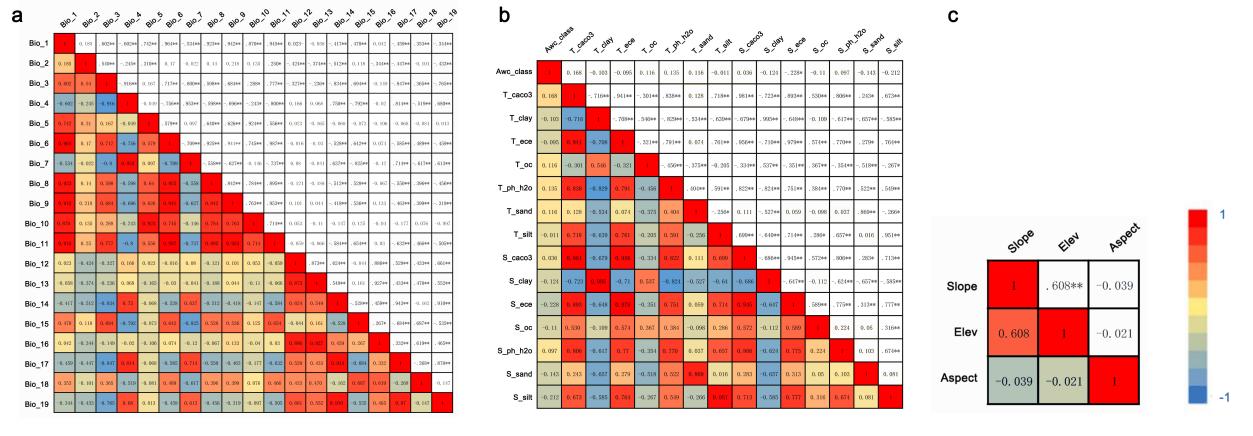


**FIGURE S1.** (a) Correlation analysis of 19 bioclimatic variables. (b) Correlation analysis of 15 soil variables. (c) Correlation analysis of 3 topographic variables.


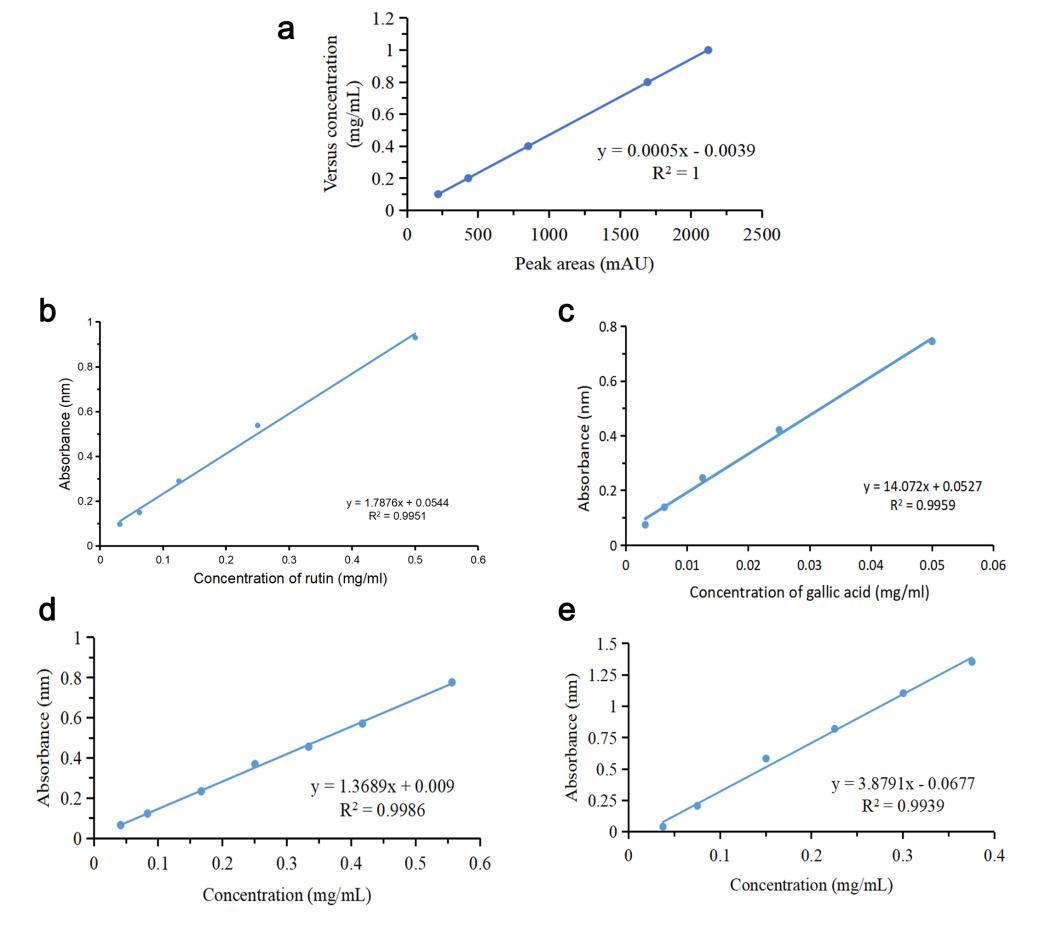


**FIGURE S2.** The standard curve. (a) mogroside V. (b) Rutin. (c) Gallic acid. (d) FeSO₄·7H₂O. (e) Trolox.


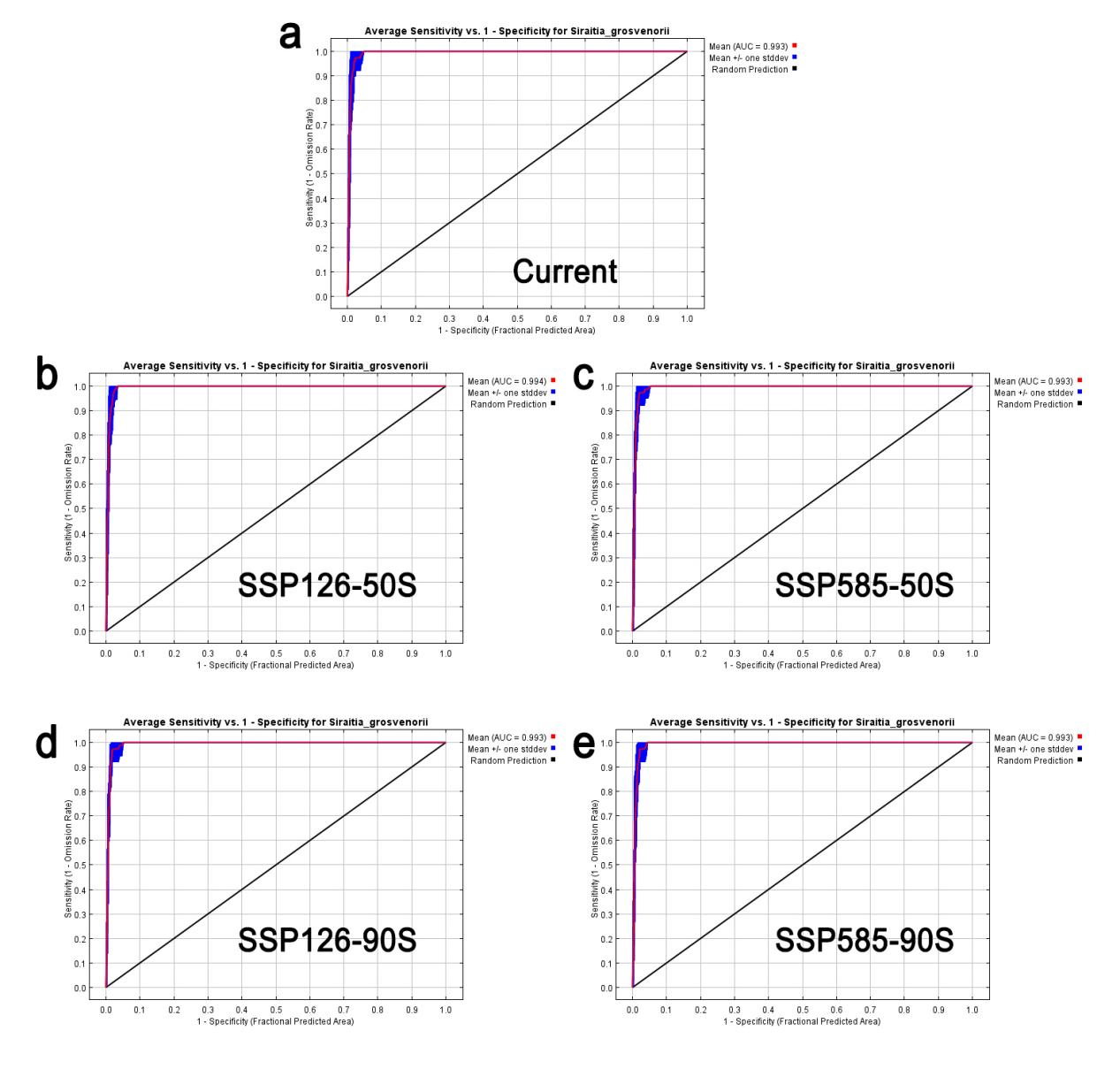


**FIGURE S3.** The average receiver operating characteristic (ROC) curve of the training set and test set of the model in different climate scenarios.


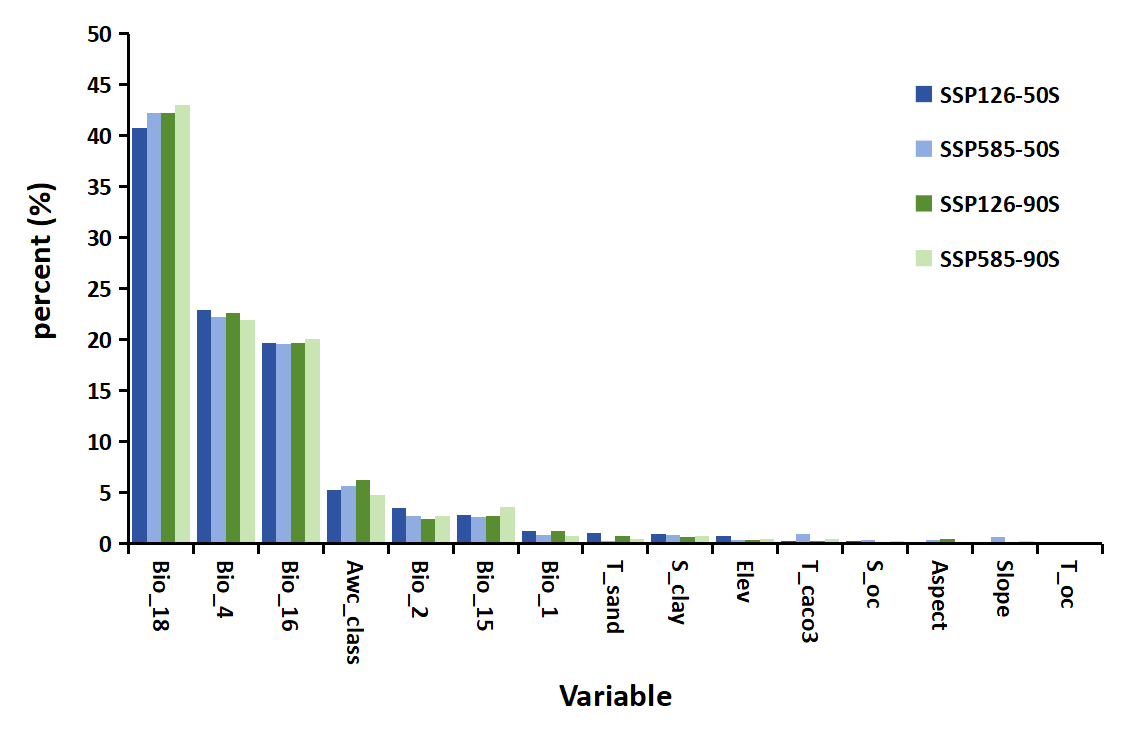


**FIGURE S4.** Percentage percent contribution and permutation importance of environment variables under different future climate scenarios.

**TABLE S1** The 76 valid occurrence records of *Siraitia grosvenorii.*

| **No.** | **Occurrence records** | **Longitude** | **Latitude** | **Specimen ID** | **Gatherer** | **Time** | **Information sources** |
| --- | --- | --- | --- | --- | --- | --- | --- |
| 1 | Emei Mount, Emeishan, Sichuan, China | 113°16’E | 23°8’N | ZMNH0042643 | Unknown | 197505 | NSII |
| 2 | Nanchuan, Chongqing, China | 107°6’E | 29°9’N | 00174299 | Huayu Zhang | 201606 | NSII |
| 3 | Jinggang Mountains, Jiangxi, China | 114°8’E | 26°38’N | 00036066 | Guanghua Sun | 201107 | NSII |
| 4 | Xunwu, Jiangxi, China | 115°39’E | 24°58’N | CZH0008244 | Xianfeng Zeng | 201304 | NSII |
| 5 | Qiyun Mount, Chongyi, Jiangxi, China | 113°16’E | 23°8’N | 00056867 | Protection zone 0701 | 200810 | CVH |
| 6 | Qianshan, Jiangxi, China | 117°42’E | 28°19’N | 0543442 | Shukun Lai, Minxiang Nie | 195808 | NSII |
| 7 | Hualian, Taiwan, China | 121°28’E | 24°2’N | 0543439 | S.Saito | 192704 | NSII |
| 8 | Yilan, Taiwan, China | 121°38’E | 24°36’N | NAS00405895 | K.Odashima | 193406 | NSII |
| 9 | Xinning, Hunan, China | 110°51’E | 26°26’N | 0543385 | Linhan liu, Guanzhou He | 196210 | NSII |
| 10 | Sangzhi, Hunan, China | 110°10’E | 29°24’N | 0544573 | Team of xiang qian | 198308 | NSII |
| 11 | Yizhang, Hunan, China | 112°57’E | 25°24’N | JJF00023312 | Bo Liu | 201602 | NSII |
| 12 | Yanling, Hunan, China | 113°46’E | 26°29’N | SYS00166821 | Yu Liu, Zhong Zhang, Minqi Cai, Yuxia liu | 201307 | NSII |
| 13 | Pingyang, Zhejiang, China | 120°34’E | 27°40’N | NAS00405973 | Renhua Shan | 196006 | NSII |
| 14 | Wenzhou, Zhejiang, China | 120°42’E | 28°0’N | CSH0107513 | Binjie Ge, Xin Zhong, Hui Shang, Ziyue Liu | 201511 | NSII |
| 15 | Wencheng, Zhejiang, China | 120°6’E | 27°48’N | ZMNH0042633 | Unknown | 197209 | NSII |
| 16 | Dongtou, Zhejiang, China | 121°9’E | 27°50’N | CSH0086095 | Weipeng Ku, Bin Shen, Yongxin Su | 201504 | NSII |
| 17 | Suichang, Zhejiang, China | 119°16’E | 28°35’N | CSH0155881 | Xin Zhong, Shang Qu, Shubo Huang | 201804 | NSII |
| 18 | Jiangkou, Guizhou, China | 108°50’E | 27°42’N | 23626 | Team of xiang qian | 198307 | NSII |
| 19 | Libo, Guizhou, China | 107°53’E | 25°25’N | 023515 | Yongkang Li | 198208 | NSII |
| 20 | Dushan, Guizhou, China | 107°33’E | 25°49’N | GZTM0065496 | Li Zhang | 201509 | NSII |
| 21 | Shiqian, Guizhou, China | 108°13’E | 27°31’N | GZTM0054840 | Xun Zhang | 201602 | NSII |
| 22 | Yanhe Tujia Autonomous County, Tongren, Guizhou, China | 106°22’E | 26°24’N | 522224LY0323 | Xun Zhang | 201602 | CVH |
| 23 | Xiuwen, Guizhou, China | 106°35’E | 26°50’N | GFS0015912 | Class 812, Group 8 | 198206 | NSII |
| 24 | Songtao Miao Autonomous County, Guizhou, China | 109°12’E | 28°9’N | GZTM0049566 | Jie Zhang | 201410 | NSII |
| 25 | Luodian, Guizhou, China | 106°45’E | 25°25’N | 0543390 | Team of qian nan | 195904 | NSII |
| 26 | Maguan, Yunnan, China | 104°24’E | 23°1’N | 0543402 | Xitao Cai | 193303 | NSII |
| 27 | Jinghong, Yunnan, China | 100°48’E | 22°1’N | 0543407 | Qiwu Wang | 193608 | NSII |
| 28 | Tengchong, Yunnan, China | 98°29’E | 25°9’N | 0543425 | Unknown | 198004 | NSII |
| 29 | Menghai, Yunnan, China | 100°27’E | 21°57’N | 0543404 | Qiwu Wang | 193606 | NSII |
| 30 | Yunlong, Yunnan, China | 99°22’E | 25°53’N | 0543413 | An expedition of aromatic plants | 198406 | NSII |
| 31 | Malipo, Yunnan, China | 104°42’E | 23°7’N | 0543415 | Qiwu Wang | 194002 | NSII |
| 32 | Funing, Yunnan, China | 105°38’E | 23°37’N | 0543410 | Sugong Wu | 196108 | NSII |
| 33 | Hekou Yao Autonomous County, Yunnan, China | 103°56’E | 22°32’N | 0543423 | Yuemei Hu, Zhaokang Wen | 195805 | NSII |
| 34 | Gongshan Dulong Nu Autonomous County, Yunnan, China | 98°40’E | 27°44’N | 0543429 | Expedition team of Dulong River | 199105 | NSII |
| 35 | Guangnali Mengla County, Xishuangbanna Prefecture, Yunnan, China | 101°33’E | 21°34’N | 01514240 | Expedition team | 198404 | CVH |
| 36 | Danzhou, Hainan, China | 109°35’E | 19°31’N | NAS00405965 | Tsang,Wai-Tak | 192804 | NSII |
| 37 | Chengmai, Hainan, China | 110°1’E | 19°44’N | NAS00405971 | Lei,C.I. | 193307 | NSII |
| 38 | Yazhou, Hainan, China | 109°10’E | 18°21’N | IBK00150615 | Kuanzhao Hou | 193303 | NSII |
| 39 | Dongle, Hainan, China | 109°11’E | 18°44’N | IBK00150632 | Xinqi Liu | 193608 | NSII |
| 40 | Baisha, Hainan, China | 109°29’E | 19°9’N | IBK00150634 | Xinqi Liu | 193603 | NSII |
| 41 | Changjiang, Hainan, China | 108°44’E | 19°17’N | IBK00150652 | Xiangri Liang | Unknown | NSII |
| 42 | Lingshui, Hainan, China | 109°54’E | 18°25’N | NAS00405968 | Tsang and Fung | 192908 | NSII |
| 43 | Haikou, Hainan, China | 110°12’E | 20°3’N | QFNU0048776 | Tong Yuan, Chengyong Guo, Yuanmian Hou, Lingjie Cao | 201804 | NSII |
| 44 | Xiamen, Fujian, China | 118°8’E | 24°30’N | AU074423 | Xueliang Hou, Wenpeng Xue | 201404 | NSII |
| 45 | Dongyuan Village, Xiandian Town, Xiangan, Xiamen, Fujian, China | 118°18’E | 24°35’N | AU078431 | Zhiwei Mao | 201909 | CVH |
| 46 | Tianzhu Mountain Forest Park, Haicang, Xiamen, Fujian, China | 117°55’E | 24°35’N | AU072437 | Yi Sheng, Yan Shi | 201903 | CVH |
| 47 | Zhangpu, Zhangzhou, Fujian, China | 117°36’E | 24°7’N | CZH0020289 | Xianfeng Zeng | 201702 | CVH |
| 48 | Zhangzhou, Fujian, China | 117°39’E | 24°31’N | 011417 | H.H.Chung | 192212 | NSII |
| 49 | Tongan, Fujian, China | 118°9’E | 24°44’N | FJIDC006224 | Unknown | 196010 | NSII |
| 50 | Xiapu, Fujian, China | 120°0’E | 26°53’N | FJIDC006175 | Jingao Lin, Youai Chen | 197808 | NSII |
| 51 | Quanzhou, Fujian, China | 118°40’E | 24°52’N | 00001100 | Hang Chi | 201311 | NSII |
| 52 | Pingtan, Fujian, China | 119°47’E | 25°30’N | 011424 | Raoting Zhang | 198007 | NSII |
| 53 | Fuzhou, Fujian, China | 119°18’E | 26°4’N | CSH0138557 | Xiangxiu Su | 201703 | NSII |
| 54 | Minhou, Fujian, China | 119°8’E | 26°9’N | CSH0140485 | Xiangxiu Su | 201701 | NSII |
| 55 | Dongshan, Fujian, China | 117°26’E | 23°42’N | 017528 | Guosheng He | 198002 | NSII |
| 56 | Zhaoan, Fujian, China | 117°10’E | 23°43’N | CZH0009522 | Xianfeng Zeng, Heyuan Qiu | 201508 | NSII |
| 57 | Yunxiao, Fujian, China | 117°20’E | 23°57’N | CZH0030468 | Xianfeng Zeng | 201705 | NSII |
| 58 | Putian, Fujian, China | 119°0’E | 25°27’N | 011426 | H.H.Chung | 192302 | NSII |
| 59 | Nanao, Guangdong, China | 117°1’E | 23°25’N | HSNU00070436 | Pengcheng Yao, Yanan Wei | 201503 | NSII |
| 60 | Jinwan, Zhuhai, Guangdong, China | 113°22’E | 22°9’N | 1485877 | Guowang Tang, Jiaxin Xi, Yanbai Pei, Mingsong Wu | 201812 | NSII |
| 61 | Guangzhou, Guangdong, China | 113°16’E | 23°8’N | AU042177 | Huanyong Chen | 193202 | NSII |
| 62 | Raoping, Guangdong, China | 116°57’E | 23°45’N | CZH0004399 | Xianfeng Zeng | 200308 | NSII |
| 63 | Fengkai, Guangdong, China | 113°16’E | 23°8’N | NAS00406001 | Cheng Huang | 195805 | NSII |
| 64 | Shunde, Guangdong, China | 113°16’E | 22°52’N | HZ021621 | Maosheng Huang | 195807 | NSII |
| 65 | Xinxing, Guangdong, China | 112°13’E | 22°42’N | HZ021622 | Yingguang LIU | 195811 | NSII |
| 66 | Zhaoqing, Guangdong, China | 112°28’E | 23°3’N | NAS00405999 | Unknown | 197604 | NSII |
| 67 | Guangning, Guangdong, China | 112°26’E | 23°38’N | 0543441 | Expedition team of Zhongyue | 196502 | NSII |
| 68 | Huadu, Guangzhou, Guangdong, China | 113°13’E | 23°24’N | 0543377 | Cheng Huang | 195808 | NSII |
| 69 | Heyuan, Guangdong, China | 114°42’E | 23°44’N | NAS00405989 | Zhaofen Wei | 195807 | NSII |
| 70 | Taishan, Guangdong, China | 112°47’E | 22°26’N | 017530 | Bangyu Chen, Zexian Li | 198011 | NSII |
| 71 | Huangmanzhai Waterfall in Jiexi County, Jieyang, Guangdong, China | 115°59’E | 23°34’N | ZXF24862 | Xianfeng Zeng | 201704 | CVH |
| 72 | Huidong, Guangdong, China | 114°43’E | 22°59’N | IBK00382475 | Zhaofen Wei | 195807 | NSII |
| 73 | Chaozhou, Guangdong, China | 116°37’E | 23°39’N | CZH0014600 | Xianfeng Zeng | 201610 | NSII |
| 74 | Fengshun, Guangdong, China | 116°11’E | 23°44’N | CSFI024502 | Xuegen Li | 195805 | NSII |
| 75 | Meixian, Meizhou, Guangdong, China | 116°9’E | 24°16’N | SN006291 | Quanfang Zhu, Xiaoan Zhang, Qineng Qiu | 198411 | NSII |
| 76 | Boluo, Guangdong, China | 114°17’E | 23°10’N | SN006287 | Nianqu Chen | 193005 | NSII |

NSII: National Specimen Information Infrastructure. CVH: Chinese Virtual Herbarium

**TABLE S2** The 37 environmental variables from WorldClim and Harmonized World Soil Database. And the percentage percent contribution and permutation importance of environment variables under current climate scenarios.

| **Variable** | | **Description** | **Percent contribution**（%） | **Permutation importance (%)** | **UNITS** |
| --- | --- | --- | --- | --- | --- |
| Bioclimatic variables | bio_18 | Precipitation of warmest quarter | 41.1 | 0.2 | mm |
|  | bio_16 | Precipitation of wettest quarter | 21.1 | 15.3 | mm |
|  | bio_4 | Temperature seasonality | 17.6 | 0.5 | ℃ |
|  | bio_14 | Precipitation of driest month | 9.4 | 4.2 | mm |
|  | bio_15 | Precipitation seasonality | 6.5 | 1.5 | mm |
|  | bio_1 | Annual mean temperature | 1.3 | 68.7 | ℃ |
|  | bio_2 | Mean diurnal range | 0.8 | 0.1 | 1 |
|  | bio_6 | Min temperature of coldest month | 0.4 | 0.2 | ℃ |
|  | bio_7 | Temperature annual range | 0.3 | 6.1 | 1 |
|  | bio_9 | Mean temperature of driest quarter | 0.3 | 0.9 | ℃ |
|  | bio_19 | Precipitation of coldest quarter | 0.2 | 0.2 | mm |
|  | bio_11 | Mean temperature of coldest quarter | 0.2 | 0.1 | ℃ |
|  | bio_3 | Isothermality | 0.2 | 1.1 | 1 |
|  | bio_10 | Mean temperature of warmest quarter | 0.1 | 0.1 | ℃ |
|  | bio_12 | Annual precipitation | 0.1 | 0.3 | mm |
|  | bio_13 | Precipitation of wettest month | 0.1 | 0.1 | mm |
|  | bio_8 | Mean temperature of wettest quarter | 0.1 | 0.1 | ℃ |
|  | bio_17 | Precipitation of driest quarter | 0.1 | 0.2 | mm |
|  | bio_5 | Max temperature of warmest month | 0.1 | 0.1 | ℃ |
| Soil variables | awc_class | Soil available water content | 15.3 | 5.4 | 1 |
|  | s_clay | Substratesoil clay content | 13.9 | 0.5 | % weight |
|  | s_oc | Substratesoil organic carbon | 11.8 | 32.7 | % weight |
|  | t_caco3 | Topsoil carbonate or lime content | 9 | 6.7 | % weight |
|  | t_oc | Topsoil organic carbon | 8.9 | 19 | % weight |
|  | t_clay | Topsoil clay content | 8.2 | 0.1 | % weight |
|  | s_ph_h2o | Substratesoil pH | 8.1 | 6.7 | 1 |
|  | t_ece | Topsoil electroconductibility | 7.5 | 1.9 | s/m |
|  | t_sand | Topsoil sand content | 6.4 | 0.2 | % weight |
|  | s_ece | Substratesoil electroconductibility | 5.5 | 1.8 | s/m |
|  | s_silt | Substratesoil silt content | 4.1 | 20.3 | % weight |
|  | s_caco3 | Substratesoil carbonate or lime content | 0.5 | 0.1 | % weight |
|  | t_silt | Topsoil silt content | 0.4 | 3.6 | % weight |
|  | t_ph_h2o | Topsoil pH | 0.3 | 0.8 | 1 |
|  | s_sand | Substratesoil sand content | 0.1 | 0.2 | % weight |
| Topographic variables | slope | Slope | 46.9 | 35.5 | ° |
|  | elev | Elevation | 45.4 | 53.8 | m |
|  | aspect | Aspect | 7.7 | 10.7 | ° |

**TABLE S3** Maximum test sensitivity plus specificity (MTSPS) of different climate scenarios.

| Periods | Current | SSP126-2050S | SSP585-2050S | SSP126-2090S | SSP585-2090S |
| --- | --- | --- | --- | --- | --- |
| MTSPS | 0.2754 | 0.2441 | 0.2172 | 0.2007 | 0.1991 |

**TABLE S4** Area of suitable habitat for *Siraitia grosvenorii* under different climate scenarios.

|  | **Low-suitable habitats** | **Medium-suitable habitats** | **High-suitable habitats** | **Total suitable habitats** |
| --- | --- | --- | --- | --- |
| Current | 5814630.65 | 3515298.12 | 533261.66 | 4048559.78 |
| SSP126-2050S | 7655243.47 | 3834968.42 | 1500873.54 | 5335841.96 |
| SSP585-2050S | 6936343.68 | 6314384.27 | 3142588.36 | 9456972.63 |
| SSP126-2090S | 8655467.46 | 4737893.65 | 1981095.73 | 6718989.38 |
| SSP585-2090S | 6187698.79 | 5866057.43 | 8271289.71 | 14137347.13 |
